# Holistic modelling as a catalyst for effective obesity policy

**DOI:** 10.1136/bmj-2023-077139

**Published:** 2024-09-10

**Authors:** Joanna McLaughlin, Carlos Sillero Rejon, Mike Bell, Bjoern Schwander, Karen Coulman, Hugh McLeod

**Affiliations:** 1 Bristol Medical School, University of Bristol, Bristol, UK; 2 National Institute for Health and Care Research, Applied Research Collaboration West, Bristol, UK; 3 Public representative, UK; 4 Agency for Health Economic Assessment and Dissemination, Bietigheim-Bissingen, Germany; 5 North Bristol NHS Trust, Bristol, UK

## Abstract

Reducing the prevalence of obesity requires multifaceted intervention, and system-wide modelling would support a move away from current piecemeal policy making towards an equitable and cost effective strategy, argue **Joanna McLaughlin and colleagues**

Despite longstanding government rhetoric of a commitment to tackling obesity, UK policies have not provided an adequate and coherent response. A 2021 analysis of 14 key obesity policy documents since 1992 identified repeated inadequacies in policy design, implementation, and evaluation.^1^ The 2023 Institute for Government report on tackling obesity concluded that the government had no serious plan to meet the aim of tackling obesity and called for learning from past mistakes.^2^

Obesity is one of the biggest health challenges of our age; prevalence in England continues to rise and reached 26% in 2021 (32% of those aged 55-74), one of the highest rates worldwide, and shows stark inequalities by deprivation.^3^ It is well established that population obesity is not going to be solved through treatment or simplistic encouragement for individuals to eat less and move more.^2^ As a report from the UK Government Office for Science concluded in 2007, obesity is a system level problem that requires simultaneous action on multiple fronts.^4^

The World Health Organization’s 2022 plan for obesity further highlights the range of settings and approaches where action must be taken, including fiscal, regulatory, and lived environment interventions.^5^ Nevertheless, UK obesity policy includes minimal population level interventions and continues to rely on short term, individual level, treatment focused approaches^1^ ^2^ that only a small percentage of the eligible population can access.^6^ The problems in policy making on obesity are symptomatic of inadequacies across many public health policy areas,^7^ ^8^ but obesity provides a good example of the problematic influence of “nanny statism” political concerns.^1^

Current siloed and fragmented approaches to appraising policy interventions limit policy makers’ understanding of the gap between the potential effect of current policy choices and stated government aims. Economic modelling capable of comparing individual policies and combinations of policies would help identify policy strategy with sufficient breadth and depth to make a difference.

## Current appraisals are siloed and fragmented

Health economic modelling enables extrapolation of measured short term cost and outcome data of interventions and controls in specific settings to predict longer term effects and associated costs and to estimate their value for money (cost effectiveness).^9^ Modelling is already commonly used to evaluate all types of obesity interventions, from individual treatments to population-wide policies, including assessment of impact in hypothetical scenarios.^10^ ^11^ However, current approaches use a fragmentary assessment of single interventions (typically making comparisons with usual care), rather than making a more holistic assessment of a comprehensive strategy. This disconnected approach gives policy makers only limited evidence of the opportunity costs of their decisions.

For example, use of obesity drugs in England is guided by the National Institute for Health and Care Excellence (NICE), which carries out technology appraisals to assess value for money. If a medicine is recommended in a NICE technology appraisal, the NHS in England is required to provide it to patients.^12^ This appraisal system can drive scarce resources towards short term use of well defined treatment options deemed cost effective, such as novel expensive drugs, without consideration of the opportunity cost for other wider services. Multidisciplinary specialist (tier 3) weight management services remain unassessed for effectiveness or cost effectiveness and underfunded.^13^

Glucagon-like peptide-1 agonists such as semaglutide are a case in point. Expectations are high for these novel injectable drugs since NICE approved their short term use for obesity. However, their effect on weight is assumed to be lost within three years of stopping treatment, and modelling of treatment for longer than the two years currently approved has not been undertaken.^14^ This short term perspective echoes the wider challenge that specialist services, if available, can also usually be accessed only for up to two years.

Another problem with current modelling is that the valuation of health and wider benefits in appraisals is inconsistent. A lack of coordination across government departments has resulted in a higher monetary value attached to quality adjusted life years (QALYs) in the Treasury’s guidance on policy appraisal (£70 000) 15 than that used as NICE’s threshold for cost effectiveness (£20 000-£30 000)**.** 16 While in theory, these monetary values represent different measures,^17^ in practice they are used for the same purpose and there is no level playing field when assessing the value for money of interventions that affect quality of life. For example, an analysis of Transport for London’s fast food advertising ban used a monetary value for a QALY of £20 000 whereas modelling of the sugar levy on soft drinks used £60 000.^18^ ^19^

##  Current obesity strategy reflects political timidity

A fragmented and siloed policy appraisal approach allows UK governments to repeatedly present a commitment to tackling obesity while advocating an ineffectual range of policies. Lobbying pressures and short term election cycles result in the recurring advocacy of politically palatable, yet inadequate, treatment focused measures that avoid the perception of an over-reaching “nanny” state.^1^ ^2^

Consequently, UK legislation for population level benefits has been rare and had limited effects. A ban on television advertising of high fat, salt, and sugar foods in or around programmes for children was introduced in 2007. However, half the television advertising seen by children in 2015 was still for such foods or related food outlets.^20^ Further advertising regulation was proposed in 2022 but is yet to be implemented after being delayed twice.^21^

A second piece of legislation was the soft drinks industry levy in 2018, which introduced tiered tariffs on drinks with 5 g or more of total sugars per 100 mL.^22^ Economic modelling using drink purchasing data from before and after the levy was introduced estimated that the combined prevalence of overweight and obesity in children and adolescents would be reduced by a maximum of 0.7 percentage points over 10 years.^22^

While these examples are important and show that is possible to introduce bold policies, in isolation each will have only a marginal effect on obesity prevalence.^22^ Less political timidity will be required to meet the government aim, set in 2018, to halve childhood obesity rates by 2030.^23^ Government decisions to postpone commitments to restrictions on multibuy deals and further advertising restrictions for high fat, salt, and sugar foods are evidence of this timidity. The ideology of choice is used to justify overly cautious timeframes in proceeding with fiscal and regulatory policies that don’t depend on individual agency to reduce obesity prevalence.^21^ Wider modelling of the effects of interventions to reduce obesity could provide evidence to counter factors such as vested interests and political impediments that are influencing policy decisions.^24^

## Embracing the challenge of holistic, system-wide modelling

Holistic modelling that compares obesity policies individually and in combination is needed to quantify the overall effect of any policy strategy. Modelling can inform both the choice of target and associated policy innovation by estimating the scale and scope of cumulative obesity policies required to achieve specific targets. Government commitment to measurable, time defined, targets for reducing obesity prevalence is therefore essential to drive progress.^2^ ^5^

Modelling would also indicate what level of spending on interventions to tackle obesity would represent good value for money compared with other uses in terms of effects on health and society. Including health inequalities in modelling would allow policy makers to decide whether to use targeted interventions for obesity that address wider determinants of health, even if these may not be highly valued in cost effectiveness terms.

There are significant challenges in implementing a holistic modelling approach. Obesity is a chronic, relapsing health condition with health and wider impacts throughout the life course. Modelling must therefore use a lifetime horizon to estimate the cumulative effect of both sustained treatment and preventive interventions.^10^ Moreover, model techniques need to be appropriate to accommodate obesity’s multiple causes and consequences.^25^ Current limitations in modelling of obesity need attention to make holistic modelling viable and ensure the effects on health are not underestimated or overestimated.

Models must avoid unwarranted assumptions, including heroic assumptions translating short term drops in calorie intake into substantial or sustained reductions in body mass index (BMI), as appetite regulation responses can undermine dietary changes in the longer term.^26^ For example, 2019 modelling reported that a successful 5% sugar reduction programme would reduce calorie intake by 19 kcal a day and UK adult obesity by 5.5%, but included a caution that unanticipated changes in eating habits could negate these effects.^11^

Additionally, current models tend to rely on four serious health outcomes associated with obesity: cardiovascular disease, cerebrovascular disease, some cancers, and type 2 diabetes.^27^ The effects of BMI on mental health, musculoskeletal health, and productivity are often missing from current models,^27^ which may considerably underestimate the cost effectiveness of interventions.^28^ An evaluation of Transport for London’s ban on fast food advertising^18^ suggested that the largest cost savings (29% of the total) resulted from prevention of osteoarthritis, and UK productivity losses related to obesity in 2021 were estimated to be £8.9bn.^29^

Problems also exist with the availability and quality of data on a breadth of long term health and societal outcomes. Potential solutions include creation of frameworks for minimum data requirements in trials that are designed to support modelling of more comparable lifetime appraisals of interventions. Increased use of natural experimental studies— in which real world data are collected from settings that have introduced policies at different times or levels of intensity—can also provide data to inform modelling of combinations of population level measures.^30^

## Towards more effective policy 

Modelling inevitably represents a simplification of reality. Nevertheless, it is a valuable tool for informing policy development and evaluation,^14^ and countering lobbying interests resisting changes to the policy status quo.^16^ A key benefit of modelling is that it can identify and explore uncertainty through transparent statistical methods to inform risk management by decision makers. Developments in microsimulation modelling techniques, and adherence to existing expert recommendations on obesity modelling, including requirements for model validation, already provide a functional framework.^10^

A more comprehensive modelling approach would highlight to policy makers, and those responsible for scrutiny, where the cumulative impact of existing policies is not sufficient. Politicians may then be emboldened to champion identified policy strategies to reduce obesity prevalence, including tackling the upstream influences on obesity.^2^ ^5^ An analogous case is the UK’s net zero strategy for greenhouse gas emissions, which took a systems approach, using modelling to work backwards from a target, rather than leaving governments to pick and choose from a “shopping list of policies which might help” 2. A similar approach for obesity would address the current bias towards individual level policies shaped by political ideology and support the acceleration of fiscal and regulatory measures that modelling is likely to show are essential for sufficient impact.^2^

The UK also needs greater clarity on the organisational responsibility and accountability for government-wide obesity policy development and implementation. After Public Health England was abolished in 2021, the Office for Health Improvement and Disparities (OHID) became responsible for leading action for healthy weight in England.^31^ However, its recent fragmentation and large cuts to staffing raise serious questions about its ability to ensure systematic public health leadership and advocacy and oversee obesity policy making.^32^

NICE has a key role in appraising interventions, and its established expertise in using modelling to inform cost effectiveness assessments makes it a natural home for the development of holistic modelling tools. The increased prominence of public health in the current consultation over NICE’s transformation is encouraging,^33^ but a fully holistic policy appraisal and advisory remit would be a substantial step away from its current function. An effective partnership with OHID, therefore, will be required to achieve a prioritisation of holistic obesity policy making, drawing on public health leadership to incorporate policy development and implementation expertise.^2^ This will also require cross government collaboration and political leadership, for which there is precedent.^34^ The Health Select Committee’s scrutiny of government decision making should use the results of modelling of policy choices.

Although holistic modelling should have a fundamental role in obesity policy innovation, it is by no means the only tool required. For example, qualitative analysis of stakeholder insight, including the experience of people living with obesity, is essential and can inform modelling parameters, validate model output, and contribute to holistic decision making. Challenging decisions lie ahead once modelling output is available—for example, the judgments to be made between policies that seek to achieve universal impact versus those targeted on health inequalities in certain population groups. Nevertheless, the ability to make such decisions based on data is essential to support government commitment to obesity policies of sufficient breadth and scope, including use of politically sensitive fiscal and regulatory measures, responsive to both cost effectiveness and health inequalities.

**Key messages**

UK policy making to reduce obesity prevalence is hindered by evaluation of interventions in isolation
Holistic, systems-wide modelling of policy strategy would highlight the combinations of policies required to meet targets and help counter barriers to action
Modelling would support scrutiny of the cost effectiveness and health inequality impacts of policy choices

## Notes

Contributors and sources: JM is a public health doctor with an interest in obesity and healthcare public health. HM and CSR are health economists with expertise in modelling and UK healthcare and public health intervention evaluation. MB is an experienced public contributor with an interest in the topic of obesity. BS has led key evidence synthesis work on this topic. KC is an NHS dietitian and clinical lecturer with expertise in weight management provision. JM and HM initiated this article after noting the challenges in identifying models to use in the evaluation of obesity interventions. JM prepared the first draft, all coauthors reviewed, edited, and approved the final draft. HM is the guarantor. We thank the BMJ reviewers and editors, whose insightful and constructive comments were of great benefit to the article.

Patient and public involvement: MB is a coauthor in the role of the public contributor. MB’s expertise allowed emphasis on the priority topics within obesity intervention from a patient and public perspective and edited the article with regard to the scientific level of language.

## References

[1] Theis DRZ White M . Is obesity policy in England fit for purpose? Analysis of government strategies and policies, 1992-2020. Milbank Q 2021;99:126-70. 10.1111/1468-0009.12498 33464689 PMC7984668

[2] Metcalfe S, Sasse T. Tackling obesity: improving policy making on food and health. 2023. https://www.instituteforgovernment.org.uk/publication/tackling-obesity

[3] NHS England. Health Survey for England, 2021 part 1. 2022. https://digital.nhs.uk/data-and-information/publications/statistical/health-survey-for-england/2021/overweight-and-obesity-in-adults

[4] Foresight. Tackling obesities: future choices. 2007. https://assets.publishing.service.gov.uk/media/5a759da7e5274a4368298a4f/07-1184x-tackling-obesities-future-choices-report.pdf 10.1111/j.1467-789X.2007.00344.x 17316292

[5] World Health Organization. WHO acceleration plan to stop obesity. 2022. https://www.who.int/publications/i/item/9789240075634

[6] Coulman KD Margelyte R Jones T . Access to publicly funded weight management services in England using routine data from primary and secondary care (2007-2020): An observational cohort study. PLoS Med 2023;20:e1004282. 10.1371/journal.pmed.1004282 37769031 PMC10538857

[7] Lewis T, Buck D, Wenzel L. Equity and endurance: how can we tackle health inequalities this time? 2022. https://www.kingsfund.org.uk/insight-and-analysis/long-reads/how-can-we-tackle-health-inequalities

[8] Barr B Higgerson J Whitehead M . Investigating the impact of the English health inequalities strategy: time trend analysis. BMJ 2017;358:j3310. 10.1136/bmj.j3310 28747304 PMC5527348

[9] National Institute for Health and Care Excellence. NICE health technology evaluations: the manual (PMG36). 2022. www.nice.org.uk/process/pmg36

[10] Schwander B Nuijten M Hiligsmann M . Identification and expert panel rating of key structural approaches applied in health economic obesity models. Health Policy Technol 2020;9:314-22 10.1016/j.hlpt.2020.03.005 .

[11] Amies-Cull B Briggs ADM Scarborough P . Estimating the potential impact of the UK government’s sugar reduction programme on child and adult health: modelling study. BMJ 2019;365:l1417. 10.1136/bmj.l1417 30996021 PMC6468887

[12] Balogan B. House of Commons Library Research Briefing: obesity policy in England. 2023. https://researchbriefings.files.parliament.uk/documents/CBP-9049/CBP-9049.pdf

[13] Public Health England. National mapping of weight management services Provision of tier 2 and tier 3 services in England. 2015. https://assets.publishing.service.gov.uk/government/uploads/system/uploads/attachment_data/file/484115/Final_Weight_Management_Mapping_Report.pdf

[14] National Institute for Health and Care Excellence. Semaglutide for managing overweight and obesity: NICE Technology appraisal guidance [TA875]. 2023. www.nice.org.uk/guidance/ta875 40080622

[15] HM Treasury. Government finance function. Guidance: the green book (2022). 2024. https://www.gov.uk/government/publications/the-green-book-appraisal-and-evaluation-in-central-government/the-green-book-2020

[16] Rawlins MD Dillon A . NICE Comes of Age. Significance 2020;17:38-41 10.1111/1740-9713.01378 .

[17] Wouterse B van Baal P Versteegh M Brouwer W . The value of health in a cost-effectiveness analysis: theory versus practice. Pharmacoeconomics 2023;41:607-17. 10.1007/s40273-023-01265-8 37072598 PMC10163089

[18] Thomas C Breeze P Cummins S Cornelsen L Yau A Brennan A . The health, cost and equity impacts of restrictions on the advertisement of high fat, salt and sugar products across the transport for London network: a health economic modelling study. Int J Behav Nutr Phys Act 2022;19:93. 10.1186/s12966-022-01331-y 35897072 PMC9326956

[19] Cobiac L Law C Smith R . Population health and health sector cost impacts of the UK Soft Drinks Industry Levy: a modelling study. [Preprint.] medRxiv 2023.10.05.23296619; 10.1101/2023.10.05.23296619 41351382

[20] Griffith R, O’Connell M, Smith K, et al. Children’s exposure to TV advertising of food and drink. Institute for Fiscal Studies, 2018. https://ifs.org.uk/sites/default/files/output_url_files/BN238.pdf

[21] Prime Minister’s Office. PM backs public’s right to choose with delay to BOGOF restrictions. Press release, 17 Jun 2023. https://www.gov.uk/government/news/pm-backs-publics-right-to-choose-with-delay-to-bogof-restrictions

[22] Cobiac LJ Rogers NT Adams J . Impact of the UK soft drinks industry levy on health and health inequalities in children and adolescents in England: an interrupted time series analysis and population health modelling study. PLoS Med 2024;21:e1004371. 10.1371/journal.pmed.1004371 38547319 PMC11008889

[23] Department of Health and Social Care. Childhood obesity: a plan for action. Chapter 2. 2018. https://www.gov.uk/government/publications/childhood-obesity-a-plan-for-action-chapter-2

[24] Anderson P Stockwell T Natera G Kaner E . Minimum unit pricing for alcohol saves lives, so why is it not implemented more widely? BMJ 2024;384:e077550. 10.1136/bmj-2023-077550 38471733

[25] Breeze PR Squires H Ennis K . Guidance on the use of complex systems models for economic evaluations of public health interventions. Health Econ 2023;32:1603-25. 10.1002/hec.4681 37081811 PMC10947434

[26] Hall KD Schoeller DA Brown AW . Reducing calories to lose weight. JAMA 2018;319:2336-7. 10.1001/jama.2018.4257 29896621

[27] McLaughlin J, Sillero-Rejon C, Moore T, et al. 19th UK Society for Behavioural Medicine annual scientific meeting: Abstract booklet. 2024. https://az659834.vo.msecnd.net/eventsairwesteuprod/production-delegatereg-public/6fbe9708e0e94bee81fb771f91093a5e

[28] Harrison S Dixon P Jones HE Davies AR Howe LD Davies NM . Long-term cost-effectiveness of interventions for obesity: a mendelian randomisation study. PLoS Med 2021;18:e1003725. . 10.1371/journal.pmed.1003725 34449774 PMC8437285

[29] Tony Blair Institute for Global Change. Unhealthy numbers: the rising cost of obesity in the UK. 2023. https://www.institute.global/insights/public-services/unhealthy-numbers-the-rising-cost-of-obesity-in-the-uk

[30] Craig P Campbell M Bauman A . Making better use of natural experimental evaluation in population health. BMJ 2022;379:e070872. 10.1136/bmj-2022-070872 36280251 PMC7613963

[31] Iacobucci G . Public Health England is axed in favour of new health protection agency. BMJ 2020;370:m3257. 10.1136/bmj.m3257 32816824

[32] West D. National agency ‘decimated’ in DHSC restructure. *Health Service Journal* 2024 Feb 8. https://www.hsj.co.uk/policy-and-regulation/exclusive-national-agency-decimated-in-dhsc-restructure/7036541.article

[33] National Institute for Health and Care Excellence . Transforming NICE. 2023. https://indepth.nice.org.uk/transforming-nice/index.html

[34] Panchamia N, Thomas P. Public service agreements and the prime minister’s delivery unit. 2014. https://www.instituteforgovernment.org.uk/sites/default/files/case%20study%20psas.pdf

